# Port-a-Cath Insertion in Pediatric Patients With Malignancy in Tabuk

**DOI:** 10.7759/cureus.17379

**Published:** 2021-08-23

**Authors:** Asmaa S Ghmaird, Mohammad S Mohammad Alnoaiji, Yousef S Alalawi, Tahani N Alrashidi, Sawsan M Al Blewi, Nagwa G Gad, Eid H Alshahrani

**Affiliations:** 1 Department of Pediatrics, University of Tabuk, Tabuk, SAU; 2 Department of Surgery, King Salman Armed Forces Hospital, Tabuk, SAU; 3 Otolaryngology - Head and Neck Surgery, University of Bisha, Bisha, SAU

**Keywords:** pediatric oncology, port-a-cath, malignancy, chemotherapy, children, complications, leukemia

## Abstract

Objectives

A port-a-cath has become the cornerstone of supportive care and therapy for most childhood malignancies. It is routinely used in children for recurrent blood sampling or intravenous therapies. This study aimed to investigate the complications of port-a-cath insertion in children, the reasons for its removal or reinsertion, and to compare open and percutaneous techniques of insertion in pediatric patients with cancer in the northwest region of Saudi Arabia.

Materials and methods

This is a retrospective observational study, which reviews pediatric cases that underwent port-a-cath insertion between 2008 and 2017. Their medical records were assessed for patient characteristics, indications for insertion, the nature of port use, their reasons for removing them, and port-related complications.

Results

We included 64 patients who had a total of 79 port-a-cath insertions in this study. The median age at first insertion was 38 months (51.56% female, 48.44% male). The mean duration between the first insertion and the removal of the port-a-cath was 36 ± 17 months. The right internal jugular vein was used in most cases. The rate of complications at our institution was 9.38%.

Conclusions

In pediatric cancer patients, a port-a-cath can be safely used, is associated with minimal complications, and can be easily managed without serious complications. The most common complications were attributed to infections, followed by the malfunction and obstruction of ports.

## Introduction

Malignancy is considered a major problem affecting children globally; it remains one of the leading causes of mortality. Approximately 400,000 children are diagnosed annually with malignancies [1]. In the United States (US), heart disease is the leading cause of death, followed by malignancy [2]. A study in the US estimated that deaths due to malignancy in children aged 0-14 years in 2018 were approximately 1,180 [3]. Leukemias, brain tumors, lymphomas, and solid neoplasms such as neuroblastoma and Wilms tumors are considered the major childhood malignancies worldwide [1]. The incidence of cancer in children less than 20 years of age accounts for 15.3 per 100,000 cases per year [4]. Cancer in children can often be treated with aggressive chemotherapy and enhanced supportive care [5].

Vascular access devices (VADs) are an essential component of health care for pediatric oncology patients. A wide range of peripheral and central venous devices provide a route to administer chemotherapy. Underestimating the importance of VAD selection can lead to the insertion of an inappropriate device, which reduces treatment efficiency and increases the risk of complications [6]. Peripheral venous devices, the tips of which do not reach the central veins of the thorax, such as conventional peripheral intravenous lines and midline peripheral lines are not recommended for the administration of chemotherapy as it could harm the peripheral veins [7]. Central venous devices are central lines that terminate in the veins within the thorax. Central lines are either peripherally inserted or centrally inserted central devices [7].

Peripherally inserted central catheters (PICCs) are most commonly inserted via the basilic, brachial, or cephalic veins. Insertion is easier and safer than centrally inserted catheters without the attendant risk of pneumothorax and hemothorax. PICCs are indicated in patients requiring several weeks to six months of IV therapy. However, compared to centrally inserted central devices, PICCs had higher rates of device-related complications as well as overall infection, thrombosis, and occlusion [8].

Centrally inserted central catheters are preferably inserted via the internal and external jugular veins. Non-tunneled, skin-tunneled, and implantable ports are the three main types of centrally inserted catheters [7]. Non-tunneled catheters can provide up to five lumens for separate access. But these lines are primarily used for five to seven days access and are typically meant for rapid resuscitation in the emergency department, operating room, and ICU. These catheters are associated with a higher risk of infection and are inappropriate for long-term access [7].

Skin-tunneled catheters, such as Hickman™, Broviac™, or Groshong™ catheters (BD Corporate, Franklin Lakes, NJ), are appropriate for long-term access and reduce the incidence of infection. These lines are preferred in patients requiring frequent, long-term access and particularly for those who need infusion of blood products [7]. The implantable port consists of a catheter attached to a reservoir that is implanted into a surgically created pocket on the chest wall or upper arm. A needle is inserted through the port’s septum to access the reservoir. Advantages include less interference with daily activities and less frequent flushing, Disadvantages include the need for needle insertion and the risk of extravasation. These devices are expensive and are more difficult and time-consuming to insert and remove than other types of devices [7]. There is a higher risk of overall infection in implantable ports compared to skin-tunneled catheters [8]. In terms of general clinical performance, there is no significant difference between skin-tunneled catheters and implantable ports. However, international guidelines recommend the use of skin-tunneled catheters for pediatric patients undergoing hematologic and oncological treatment [8,9].

A port-a-cath is a device used to administer chemotherapy, but it can also be used to transfuse blood products, initiate antibiotics and IV fluids directly into the bloodstream, and draw blood samples [10,11]. It may be inserted subcutaneously in the tissue of the upper chest [10]. Many chemotherapy treatments are administered through a large vein because they may cause severe irritation phlebitis, urticaria, vasospasm, pain, and chemical burns if administered through the peripheral veins [5,11].

A port-a-cath is effective for cancer patients who require a long period of IV treatment and blood sampling, because it enhances the quality of life, improves daily hygiene, and does not interfere with other activities [11,12]. However, many complications may still result from the use of a port-a-cath, such as infection, occlusion, thrombosis, skin necrosis, extravasation, migration, and dislodgment. The dislodgment of a port-a-cath is rare but life-threatening; it may cause arrhythmia, heart or vessel perforation, cardiac tamponade, and even death [13,14]. However, there are a limited number of studies addressing port-a-cath insertion and its complications in our region. We aim to report our experiences with port-a-cath insertion in children, its complications, and to compare open versus percutaneous techniques.

## Materials and methods

This is a retrospective observational study that reviewed medical records of pediatric patients with malignancies who had a port-a-cath insertion between 2008 and 2017 at King Salman Armed Forces Hospital (KSAFH)’s oncology center. A total of 79 port-a-caths were inserted in 64 out of 71 patients with malignancy at our institution.

Ethical approval was obtained from the KSAFH Research Ethics Committee. We included all children with malignancy who underwent port-a-cath insertion at < 13 years of age. We excluded children with malignancy who did not undergo port-a-cath insertion, children who had undergone port-a-cath insertion but did not have cancer, children over the age of 13, and those with missing information.

The following data were collected from the patients’ medical records: a) age at insertion and removal; b) sex; c) duration; d) the laterality of the port-a-cath (right, left); e) location of insertion (internal jugular vein (IJV) and external jugular vein); f) type of surgical technique (open or percutaneous); g) reasons for removal; h) complications; and i) reinsertion and reasons for reinsertion. Each patient was given an identifier code in the Microsoft Excel (Microsoft Corp., Redmond, Washington) datasheet to ensure patient confidentiality. 

Surgical technique

All children who undergo port-a-cath insertion are put under general anesthesia. They receive prophylactic antibiotics prior to the procedure. A 2.5-mL syringe is used to flush the needle with heparinized saline. Sites of insertion include the subclavian, internal jugular, and external jugular veins.

The participants were subdivided into two groups: group 1 (open technique) with 25 members, and group 2 (percutaneous technique) with 39 members, respectively. We prefer the US-guided percutaneous over the open technique and it is the technique of choice in our hospital because it is less invasive, does not involve any neck incision nor neck dissection (more cosmetic), besides being faster and safe. The IJV is the site of choice for the insertion of central lines in our hospital, preferably performed on the right side, where the pathway to the right atrium is straight, and there is virtually no chance of thoracic duct injury.

The IJV can be accessed percutaneously or by an open technique. The open technique lasts 10-15 minutes longer than the percutaneous technique with an incision that is bigger by 1cm; besides, the percutaneous technique is more cosmetic with fewer complications and less bleeding. On the other hand, the open technique requires only one C-arm x-ray, while the percutaneous technique needs at least three.

Statistical analysis

The IBM SPSS software was used for all statistical analyses. The categorical variables were described using frequencies and absolute numbers. The data with a normal distribution are displayed as proportions (mean ± SD) and range, while data that do not conform to a normal distribution are displayed as the median.

## Results

A total of 79 port-a-caths were inserted in 64 children with malignancy at our institution. Of the 64 patients, 33 were female (n=33; 51.56%) while 31 were male (n=31; 48.44%). The median age at the first port-a-cath implantation was 38 months, with a range between five months and 12 years.

The right IJV was the most common site for catheter implantation (n=56; 87.50%), followed by the left IJV (n=7; 10.94%), and only one case in the right external jugular vein. Sixty-four patients had 79 port-a-caths inserted; 15 patients had to have ports reinserted in a second time: 10 patients due to relapse and five due to port-a-cath-related complications.

The patients who underwent first-time port-a-cath removal were 58 out of 64. The rest had their catheters in at the time of this report. The mean duration of port-a-cath use was 36 ± 17 (range, 5-81) months for all children, 31 ± 22 months (range, 8-72 months) for children with port-a-cath-related complications, and 37 ± 16 (6-64) months for children without complications (Table 1).

**Table 1:** Baseline characteristics of 64 patients who underwent port-a-cath insertion.

Variables	Results
Median age at insertion, (months)	38
Range	5 months – 12 years
Sex n (%)	
Female	33 (51.56 %)
Male	31 (48.44 %)
Mean duration between first insertion and removal of porta. (Mean ± SD)	
All children	36 ± 17
With complications	31 ± 22
Without complications	37 ± 16

Children who underwent port-a-cath insertion were classified in terms of the underlying oncology diagnosis as follows: acute lymphoblastic leukemia (ALL) (n=40; 62.50%), acute myeloid leukemia (AML) (n=6; 9.38%), neuroblastoma (n=5; 7.81%), Wilms tumor (n=3; 4.69%), Ewing’s sarcoma (n=2; 3.13%), Burkitt lymphoma (n=2; 3.13%), medulloblastoma (n=2; 3.13%), yolk sac tumor (n=1; 1.56%), pilocytic astrocytoma (n=1; 1.56%), and rhabdoid tumor (sacrococcygeal tumor) (n=1; 1.56%).

The port-a-caths were removed in almost two-thirds of the patients. The reasons for this were: most of the patients (39) completed their treatment plan, 10 patients underwent bone marrow transplantation (BMT), six patients had port-a-cath-related complications, three patients died (related to primary disease), and one patient refused to continue chemotherapy (Table 2).

**Table 2:** The underlying oncology diagnosis of port-a-cath insertion and reason of removal in the open vs. percutaneous techniques. ALL: Acute lymphoblastic leukemia; AML: Acute myeloid leukemia.

Variable	Open (n=25)	Percutaneous (n=39)	Total (n=64)
Reason to remove the catheter n (%)			
Complete chemotherapy/ remission	16 (64.00)	23 (58.97)	39 (60.94)
Infection	1 (4.00)	1 (2.56)	2 (3.13)
Malfunction/ obstruction	0 (0.00)	2 (5.13)	2 (3.13)
Catheter displacement	1 (4.00)	0 (0.00)	1 (1.56)
Parents refuse treatment	1 (4.00)	0 (0.00)	1 (1.56)
Bone marrow transplantation	4 (16.00)	6 (15.38)	10 (15.63)
Death (related to primary disease)	2 (8.00)	1 (2.56)	3 (4.69)
Diagnosis n (%)			
ALL	17 (68.00)	23 (58.97)	40 (62.50)
AML	0 (0.00)	6 (15.38)	6 (9.38)
Neuroblastoma	3 (12.00)	2 (5.13)	5 (7.81)
Wilms tumor	0 (0.00)	3 (7.69)	3 (4.69)
Burkitt lymphoma	2 (8.00)	0 (0.00)	2 (3.13)
Medulloblastoma	1 (4.00)	1 (2.56)	2 (3.13)
Yolk sac tumor	1 (4.00)	0 (0.00)	1 (1.56)
Ewing Sarcoma	0 (0.00)	2 (5.13)	2 (3.13)
Osteosarcoma	1 (4.00)	0 (0.00)	1 (1.56)
Pilocytic astrocytoma	0 (0.00)	1 (2.56)	1 (1.56)
Rhabdoid tumor (Sacrococcygeal tumor)	0 (0.00)	1 (2.56)	1 (1.56)

The rate of complications at our institution was 9.38%. Postoperative complications including infection (3.13%), malfunction/obstruction of the catheter (3.13%), catheter migration (1.56%), and necrotic skin over the port-a-cath device (1.56%), in six patients. Moreover, these complications were most common in patients with ALL, followed by neuroblastoma (Table 3). 

**Table 3:** Complications of port-a-cath

	Variable	Open (n=25)	Percutaneous (n=39)	Total (n=64)
Complications n (%)	Absent	23 (92.00)	35 (89.74)	58 (90.63)
	Present	2 (8.00)	4 (10.26)	6 (9.38)
Reported Complications n (%)	Infection	1 (4.00)	1 (2.56)	2 (3.13)
	Malfunction/ obstructed	0 (0.00)	2 (5.13)	2 (3.13)
	Catheter migration	1 (4.00)	0 (0.00)	1 (1.56)
	Skin necrosis	0 (0.00)	1 (2.56)	1 (1.56)

## Discussion

Ports are commonly and safely used in children with cancer to facilitate treatment protocols. They are also routinely used in children to draw blood samples or initiate intravenous therapies [15]. Our study provides the experience of our institution with the implantation of port-a-cath and complications, as well as a comparison of both techniques of insertion.

The most widely used modality in pediatric cancer therapy is chemotherapy, which nearly always involves the combination of drugs and requires administration over a period of time. This helps patients receive multiple chemotherapies and enhances their quality of life [16]. In our study, 79 port-a-caths were inserted in 64 children with cancer. The primary underlying conditions that required chemotherapy and port-a-cath insertion were ALL followed by AML. This is similar to the findings of previous studies [5,17]. This is a contrast with the study reported by Chandrasekaran et al. that revealed the most common underlying condition was AML followed by ALL [18]. While a study in Indian reported that ALL was the most common underlying diagnosis followed by neuroblastoma [19]. This is because leukemia is the most frequent cancer in children, accounting for approximately 25% of cases [17].

In our study, none of the patients underwent subclavian vein (SCV) port-a-cath insertion. Furthermore, several studies have reported that the IJV is the most common site of insertion [5,17,19,20], which is similar to our study. This could be explained by the rates of pneumothorax, upper extremity deep vein thrombosis, and pinch-off syndrome in IJVs compared to the SCV [17]. Nonetheless, the use of a port-a-cath has been associated with many complications, but most can be managed without serious morbidity or mortality. In the present study, the rate of complications at our institution was 9.38%, which is consistent with the complication rates of previous studies (7%) [21,22].

The mean duration of the ports in this study was 36 ± 17 months. This is longer than that of a previous study, which ranged from 12 to 22 months [23]. The long duration of the ports has been associated with risk factors for infection [23]. Furthermore, the mean duration of port-a-cath use in our study was longer than that reported in previous studies. Despite the longer duration of port use, the rate of infection in our institution was 3.13%, which is lower than that reported in a previous study (16%) [23]. Moreover, this finding is consistent with the rate reported by Babu et al. (3.6%) [22]. To decrease the infection rate, healthcare providers should deliver comprehensive patient and family education, in addition to them having well-established training [23]. 

Mechanical complications were considered the second-most common port-a-cath-related complications in our study. One case each of malfunction, obstruction, and catheter separation were observed (4.69%). This is consistent with Bawazir et al., who reported a rate of 5%-7% [23]. Skin necrosis over the port-a-cath device occurred in one patient. This is also consistent with a study published in India by Aparna et al. [5]. 

BMT usually cures cancer and non-cancer diseases in children and adults [24]. It can cure malignant hematopoietic conditions [25]. It is salvage therapy for children with malignancies after receiving chemotherapy and radiation. Completion of treatment is an indication for port-a-cath removal [25]. However, removal of ports is only indicated in children who completed treatment, underwent BMT, and/or there is no further need for a port.

Sixty-four patients underwent insertion of 79 port-a-caths of which only 15 had to be reinserted. Of these 15, only five were due to port-a-cath-related complications. However, port-a-cath removal should be emphasized before the procedure, to avoid putting patients at risk. Of the 79 ports in our study, almost two-thirds underwent port removal (73.42%). Most patients, 39, had it removed on completion of their treatment.

Limitations

The limitations of our study include its retrospective and single-center design. We recommend conducting future studies with a prospective cohort design. In addition, younger children may show different physiological responses than older ones, based on the therapy, age, and body size difference between young age and older children. We thus recommended a prospective study that investigates the response of therapy according to age, body size, sex, and underlying conditions, and how these factors may affect duration of catheter.

## Conclusions

A port-a-cath can be safely used in pediatric patients with malignancies and can be easily managed without serious complications. The most common complications at our institution were infections, followed by malfunction/obstruction of ports. Complications were most commonly observed in patients with ALL, followed by neuroblastoma. The handling of port-a-cath for pediatric patients who require chronic venous access needs special care from health care providers, the patients themselves, and their families, to decrease the rate of infection.
